# Effects of FDI on the Efficiency of Government Expenditure on Environmental Protection Under Fiscal Decentralization: A Spatial Econometric Analysis for China

**DOI:** 10.3390/ijerph16142496

**Published:** 2019-07-12

**Authors:** Jie Zhang, Yinxiao Qu, Yun Zhang, Xiuzhen Li, Xiao Miao

**Affiliations:** 1 School of Business, Hohai University, West Focheng Road 8, Nanjing 211100, China; 2 Collaborative Innovation Center for Coastal Development and Preservation, Xikang Road 1, Nanjing 210098, China; 3 School of Finance, Shanghai Lixin University of Accounting and Finance, Shanghai 201620, China; 4 School of International Economy and Trade, Shanghai Lixin University of Accounting and Finance, Shanghai 201209, China; 5 Innovation Research Institute of Traditional Chinese Medicine, Shanghai University of Traditional Chinese Medicine, Shanghai 201203, China

**Keywords:** foreign direct investment, fiscal decentralization, efficiency of government expenditure on environmental protection, spatial model, spillover effect

## Abstract

Most governments strive for an ecological civilization so the efficiency of government expenditure on environmental protection (EPEE) is an important issue. While it is recognized that foreign direct investment (FDI) enhances environmental protection, this investigation focuses on the effects of FDI on the efficiency of government expenditure on environmental protection under fiscal decentralization. Analysis is conducted using an output-oriented data envelopment analysis (DEA) scale return model to calculate the efficiency of environmental protection spending in China. Then, a spatial model is built to test the linkages among FDI, fiscal decentralization and the efficiency of government expenditure. The results reveal that, firstly, the efficiency of government spending has been enhanced over the last 10 years. Secondly, FDI is positively correlated with the efficiency of government environmental expenditure in terms of both quantity and quality of spending and it has a positive spillover effect. Thirdly, financial decentralization is negatively correlated with the efficiency of environmental spending, but it improves the effect of FDI. Accordingly, policy proposals are that the government should improve the supervision system for environmental spending and local governments should pursue FDI, improve the structure of FDI and use its spillover effect to enhance the efficiency of environmental expenditure.

## 1. Introduction

Government expenditure on environmental protection is the main guarantee for achieving environmental protection and an important determining factor for environmental quality. In addition, the composition of government expenditure can be reallocated to enhance pollution abatement [1]. However, the local government faces a dilemma between developing the economy and protecting the environment because it has limited financial resources. The efficiency of expenditure constitutes an important key to understanding government expenditure on environmental protection. Furthermore, understanding how to improve the efficiency of governmental spending on environmental protection is conducive to achieving balance between economic growth and environmental protection. If local governments absorb more foreign direct investment (FDI), this enhances economic development and national income increases. Based on Wagner’s Law, government expenditure will increase by a larger proportion. According to the environmental Kuznets curve (EKC) hypothesis, the improvement of environmental quality is achieved when national income reaches a certain level [2]. Adequate fiscal funds increase environmental funds and ensure that local governments have more energy and financial freedom to govern environmental protection.

Fiscal decentralization gives local governments fiscal autonomy to allocate financial funds and this has become the modus operandi in many countries. To varying degrees, each country has experienced the decentralization of fiscal power from the central to the local government. In order to achieve rapid economic growth, local governments may engage in economic competition and develop industrial enterprises; this often means that environmental protection is neglected. Local government may not spend enough energy and money on environmental protection under fiscal decentralization. When local governments absorb FDI, the competition between governments is no longer focused on economic competition; environmental protection becomes a point of competition [3]. Surrounding regions may learn about cleaner technologies in production and imitate pollution control methods. In this paper, we strive to develop the understanding of the relationship between FDI, fiscal decentralization and the efficiency of government spending on environmental protection by considering the following research questions. Based on the quantity and quality of FDI, what is the impact of FDI on the efficiency of government spending on environmental protection? Is there a spillover effect? Does fiscal decentralization influence the effect of FDI? The remainder of this paper is organized as follows: Section 2 reviews the extant literature and Section 3 describes the measurement method for the efficiency of government expenditure on environmental protection (EPEE), as well as the spatial econometric model. Section 4 presents the sample and variables. The status quo regarding the efficiency of government environmental protection spending, test results, empirical results and robustness analysis are reported in Section 5. Section 6 draws conclusions and presents policy recommendations.

## 2. Literature Review and Research Hypotheses

### 2.1. The Efficiency of Government Expenditure on Environmental Protection (EPEE)

In terms of the efficiency of government expenditure, extant studies consider both the efficiency of overall government expenditure and the efficiency of the government expenditure on a single item. The efficiency of overall government expenditure calculated by many scholars indicates the efficiency of payment by the government in providing public goods and services to society [4,5,6,7,8,9]. Studies of the efficiency of government expenditure on a single item correspond to the public functions of local governments, such as the efficiency of government expenditure on firefighting [10], solid waste disposal [11], health [12,13], education [14], agriculture [15], the technical efficiency of public universities in New York, USA [16] and the performance of general transfer payments [17]. The efficiency of government expenditure on a single item represents the efficiency of each functional department using funds. EPEE refers to the efficiency of environmental protection departments in controlling and using fiscal expenditures [18]. This efficiency measure can reasonably capture the effective use of environmental protection funds, or lack thereof. Environmental protection funds are mostly invested in protecting environment and governing environmental pollution. Based on the input–output analysis method, EPEE becomes higher for a given level of government expenditure on environmental protection, if the pollution abatement rate in the region increases. The local government environmental expenditure for pollution abatement is the key to improving EPEE.

The most common method of analysis the efficiency of government expenditure is the data envelopment analysis (DEA) model. For example, the DEA model and the three-stage DEA model are used to analyze the efficiency of public finance expenditure [6,7]. Some scholars use the DEA–Tobit model to measure the overall government expenditure efficiency and analyze the influencing factors [8,9]. For EPEE, scholars use both the content analysis method to conduct qualitative analysis of the performance of environmental protection financial funds and quantitative analysis of EPEE to discuss influencing factors [19,20,21,22,23]. The common methods to calculate EPEE are the output-oriented DEA scale return model and the DEA–Malmquist model [18,20,21,22]. Based on the input–output analysis, the government expenditure on environmental protection is taken as an input indicator [18,20,21,22]. Some scholars select the total amount of industrial wastewater discharged, industrial exhaust gas and industrial fixed waste to represent the degree of environmental pollution in each place as output variables [21]. Some scholars take wastewater discharge, sulfur dioxide emissions, domestic waste removal volume and local afforestation area as output indicators representing local environmental pollution, local environmental governance capacity and local environmental governance results [18]. Some scholars use the pollution removal amount or removal rate indicating environmental governance capacity as expected outputs [20,22].

### 2.2. FDI and EPEE

Numerous studies about the effect of FDI on environmental protection have been conducted. Under the two opposing views of the ‘pollution heaven’ hypothesis and the ‘pollution halo’ hypothesis, there are different consequences for environmental protection when FDI increases. In the former case, pollution-intensive enterprises from developed countries influence the energy consumption structure of the host country negatively, which ultimately causes the deterioration of the host country’s environment [24]. For example, empirical research on Latin America has been conducted to verify that the increase in FDI between 1980 and 2007 increased CO_2_ emissions [25]. Scholars draw the same conclusion in their research on Thailand and India [26,27]. On the other hand, the relatively cleaner technologies in production and pollution control introduced by FDI can positively affect the environmental technologies of host countries, thus resulting in green spillover, which improve environmental quality in host countries [28,29]. For example, it was found that the increase in CO_2_ emissions stem from energy consumption and gross domestic product (GDP) growth, rather than FDI, based on research of the Gulf Cooperation countries [30]. FDI has been found to reduce the use of pollutive technology [31]. Overall, the effect of FDI on the environment is positive, negative or not obvious in developing countries [32,33].

The effect of FDI on the environment in China’s economic reform and opening-up has received extensive attention and discussion. Previous studies consider different mechanisms such as scale, technology and structure and obtain different results. FDI can be considered in terms of two aspects: Quantity and quality. The quantity of FDI is the amount of FDI absorbed and the quality of FDI is a foreign investment characteristic that reflects whether investments satisfy the needs of the recipient country and generates benefits [34]. The quantity of FDI plays a positive role in improving environmental quality in China from the perspective of geographical clustering based on spatial models [35]. The effect of FDI quality on environmental improvement has significant threshold characteristics and with the improvement of local absorption capacity, the effect of FDI quality is also enhanced [34]. Some scholars divide the environmental effect of FDI in China into three effects: The scale effect, the composition effect and the technique effect, and use the simultaneous equation model to find that the technique effect is negative, whereas the scale effect and composition effect of FDI are positive [36]. This further study is necessary to confirm that FDI plays an important role in pollution abatement, which is mainly attributed to green technologies [36]. Conflicting evidence still exists on this topic. A negative effect of FDI on China’s environmental quality, in general, has been found based on an investigation of the effect of FDI and government regulation on effluents [37]. The quality of FDI has been found to not significantly impact industrial environmental efficiency and the scale of FDI has been found to have a negative impact [38].

The improvement of the environmental effects of technologies is influenced by the economic and regulatory systems of the host country [39]. With the updating and improvement of policies, the focus of FDI has shifted from labor-intensive general manufacturing to high-tech industries, so that advanced environmental technologies have also been brought into the country. The increase in the quantity of FDI is conductive to the introduction of green technology and improvement of the quality of FDI benefit technological advancement. The advanced environmental technology has a positive impact on environmental governance, improving environmental governance capacity and enhancing environmental governance effects [36]. Stronger environmental governance capacity improves EPEE. Advanced environmental technology is also conducive to improving pollution abatement efficiency, which benefits EPEE. From the perspective of quantity and quality, technology effect from FDI quality is higher than FDI quantity because technological progress is more obvious under the requirement of FDI quality.

In addition, the increase in FDI is conducive to economic growth and government revenue. FDI significantly improves GDP growth for the developing Asian countries and affects GDP through knowledge and new technologies [40,41]. Wagner’s Law says that government expenditure will increase by a larger proportion when national income increases. The increase in government revenue is conducive to accumulating fiscal fund reserves. Sufficient fiscal funds will help the government to shift its attention from GDP growth to environmental issues. What is more, as the indicators of government performance are no longer dominated by economic development, it will help adjust the structure of regional government expenditure and increase expenditure on environmental protection. Therefore, FDI affects EPEE positively through environmental technology and the structure of regional government expenditure on environmental protection. The growth of FDI increases pollution abatement capacity and the scale of government expenditure on environmental protection. As for the spatial spillover effect, scholars have developed two theories: ‘Promotion theory’ and ‘exclusion theory’. FDI has a significant technology spillover effect in 49 countries, while the study of 44 developing countries validates the negative spillover effect of FDI [42,43]. Chinese scholars exhibit the same lack of consensus. Some scholars find that FDI promotes domestic technological progress, while other scholars argue that FDI does not influence domestic technological progress positively [44,45,46,47]. From the policy perspective, the technology spillover of FDI is the most significant in technology-intensive industries [48]. FDI shows significant positive spatial agglomeration and spillover effects [49].

New economic geography incorporates spatial clustering of economic activities into the analysis. Based on this perspective, the increase in FDI is conducive to the concentration of economic activities [50]. Advanced environmental technologies produce certain spillover benefits and affect the environmental governance capacity of surrounding areas. The technology spillover effect of FDI affects surrounding areas. The structure of regional government expenditure on environmental protection affects the local environmental management performance. The interaction of environmental protection behavior in the neighboring areas increases because of environmental degradation. Besides, local government expenditure on environmental protection have spillover effects as public goods, and more financial funds are invested in pollution-intensive industries to improve the environment of the region, which benefits the environment in neighboring regions at the same time [51].

**Hypothesis 1** **(H1).** *FDI has a significant, positive impact on EPEE.*

**Hypothesis 1a** **(H1a).** *The quantity of FDI promotes EPEE and has a positive spillover effect.*

**Hypothesis 1b** **(H1b).** *The quality of FDI promotes EPEE and has a positive spillover effect.*

### 2.3. FDI, Fiscal Decentralization and EPEE

In terms of the relationship between fiscal decentralization and environmental protection, two views can be observed in the literature. One view is that fiscal decentralization can be expected to be negatively correlated with the scale of pollutant discharge [52]. Under the centralized and decentralized government system, decentralization is conducive to the improvement of regional environmental quality [53]. Most scholars support another view and argue that fiscal decentralization leads to vicious economic competition between governments, resulting in a deterioration of environmental quality. Accordingly, fiscal decentralization has been found to improve sewage disposal and increase pollutant discharge [54,55]. Further studies find that when local governments lower the standards of environmental supervision to achieve regional economic growth and fiscal revenue because of the decentralized system, environmental quality declines [56,57,58,59]. Local governments regard economic performance as the main evaluation index so government expenditure on industrial pollution control declines and industrial pollutant discharge increases [60]. In this case, the environmental protection department plays a weak role and neglects EPEE. It is also argued that fiscal decentralization brings environmental pollution problems through the channels of tax competition [61,62,63,64,65]. These authors find that tax competition has a negative effect on the optimal allocation of regional resources and deteriorates the environmental quality of the region. The deterioration of environmental quality reflects the low EPEE.

Concerning the relationship between FDI and fiscal decentralization, most scholars argue for a positive relationship. Fiscal decentralization has a positive and significant effect on inbound FDI [49,66]. Some scholars examine the role of FDI and fiscal decentralization on green total factor productivity and find that FDI cannot effectively improve it but the positive interaction with fiscal decentralization significantly promotes the growth of green total factor productivity [67]. FDI puts higher demands on the governance efficiency and management level of government and efficient management behavior is conducive to stimulate the spillover effect of FDI [67]. The positive interaction between FDI and fiscal decentralization is conductive to the advancement of environmental technology and the adjustment of the structure of government expenditure.

**Hypothesis 2** **(H2).** *FD can improve the effect of FDI on EPEE although there is a negative relationship between FD and EPEE.*

**Figure 1:**
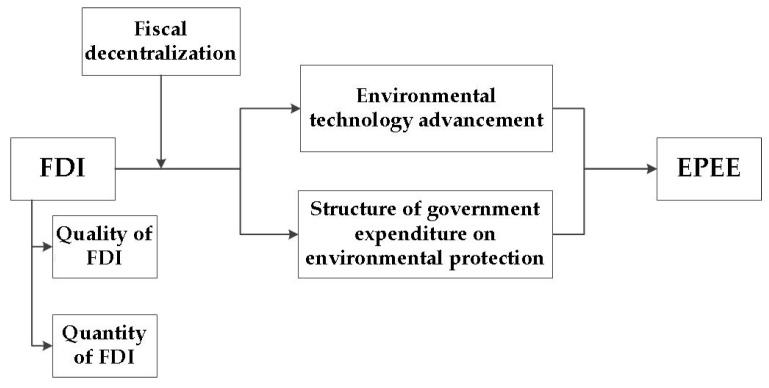
Foreign direct investment (FDI), fiscal decentralization and the efficiency of government expenditure on environmental protection (EPEE) logic diagram.

In sum, for the remainder of this article, EPEE will stand for the concept of the efficiency, that is, the value and execution, of government expenditure on environmental protection. As shown in Figure 1, FDI affects EPEE through environmental technology advancement and the structure of government expenditure on environmental protection. Fiscal decentralization is related to EPEE and influences the effect of FDI. FDI has positive spatial spillover effect. This paper will first use the DEA scale return model from the perspective of input and output to calculate EPEE scientifically and reasonably, and then use the spatial model to analyze FDI, fiscal decentralization and EPEE based on panel data from 30 Chinese provinces from 2007 to 2016.

## 3. Methodology

### 3.1. EPEE Measurement Method

The DEA model is widely applied to the efficiency assessment of the public sectors because it can effectively avoid statistical errors [68,69]. This paper selected the output-oriented DEA scale return model focusing on the extent to which each output should increase to achieve efficiency without increasing input [18,20]. The focus regarding EPEE is on the maximum output that a certain level of government expenditure can achieve, so the model is applicable to the calculation. We assumed that there were decision making units (DMUs) that consume *p* inputs to produce *q* outputs. The output-oriented DEA scale return model is as follows:

(1) $$\begin{array}{c} Max\;\Theta \\ s.t\sum_{i=1}^{n}\lambda_{i}x_{i}+s^{-}=X\\ \sum_{i=1}^{n}\lambda_{i}Y_{i}-s^{+}=\Theta Y\\ X_{i}={\left(x_{li},\ldots ,x_{pi}\right)}^{T}\\ Y_{i}={\left(y_{li},\ldots ,y_{qi}\right)}^{T}\\ \lambda \geq 0,\;i=l,\ldots ,n\\ s^{-}\geq 0,\;s^{+}\geq 0, \end{array}$$

where θ, and $s^{+}$ are the main indicators when applying the DEA scale return model to evaluate efficiency, where *θ* is the efficiency evaluation index, and are the relaxation vectors of input and output. When *θ* > 1, the corresponding DMUs are inefficient, that is, the existing input of the unit can obtain more output. When *θ* = 1, and at least one of and is not 0, then the corresponding DMUs are weakly valid. When *θ* = 1, and are both 0, the corresponding DMUs are efficient, that is, the unit cannot obtain more output through the existing input.

### 3.2. Spatial Econometric Model

As discussed above, FDI can diffuse spill over to neighboring provinces because of spatial heterogeneity and spatial correlations. Thus, the EPEE of each province is influenced by the local and neighboring province’s FDI. Therefore, we establish the spatial econometric model using a panel data set on 30 provincial-level administrative regions. We will refer to Equation (2) as the general nesting spatial (GNS) model, which contains all types of interaction effects [70].

(2) Y=ρWY+βX+θWX+u, μ=λWμ+ε

W denotes the non-negative spatial weight matrix. *WY*, *WX* and *Wμ* stand for the endogenous interaction effects of the explained variable, the exogenous interaction effects among the explanatory variables and the interaction effects among the disturbance terms of the different observations, which allow us to analyze the spillover effects of the explanatory variables. *ρ* and measure the strength of dependence between units, while *β* and *θ* are the spatial regressive coefficients. The GNS model obtains six kinds of spatial econometric models. It is argued that the spatial Durbin model (SDM) can capture spatial correlation of the explained variable and spatial spillover effects of explanatory variables so we choose the SDM to examine the effect of FDI and, fiscal decentralization (which will henceforth be referred to as ‘*FD*’) on *EPEE* [71].

To control for other influencing factors, we add environmental regulation (*ER*), the level of economic development (*EC*), total population (*POP*), energy consumption structure (*ES*) and urbanization level (*UL*) as control variables [19,22]. Based on the previous study [72], three specific econometric models are constructed. Namely:

(3) $$\begin{array}{cc} EPEE_{it}= & \rho \sum_{ij}^{N}w_{ij}EPEE_{jt}+\alpha_{1}FI_{it}+\beta_{1}\sum_{ij}^{N}w_{ij}FI_{jt}+\alpha_{2}FD_{it}\\ & +\alpha_{3}\ln{\left(ER\right)}_{it}+\alpha_{4}\ln{\left(EC\right)}_{it}+\alpha_{5}\ln{\left(POP\right)}_{it}+\alpha_{6}ES_{it}\\ & +\alpha_{7}UL_{it}+\alpha_{8}UL_{it}{}^{2}+\mu_{i}+\varepsilon_{it}, \end{array}$$

(4) $$\begin{array}{cc} EPEE= & \rho \sum_{ij}^{N}w_{ij}EPEE_{jt}+\alpha_{1}\ln{\left(SC\right)}_{it}+\beta_{1}\sum_{ij}^{N}w_{ij}\ln{\left(SC\right)}_{jt}\\ & +\alpha_{2}FD_{it}+\alpha_{3}\ln{\left(ER\right)}_{it}+\alpha_{4}\ln{\left(EC\right)}_{it}\\ & +\alpha_{5}\ln{\left(POP\right)}_{it}+\alpha_{6}ES_{it}+\alpha_{7}UL_{it}+\alpha_{8}UL_{it}{}^{2}+\mu_{i}+\varepsilon_{it} \end{array}$$

(5) $$\begin{array}{cc} EPEE_{it}= & \rho \sum_{ij}^{N}w_{ij}EPEE_{jt}+\alpha_{1}EX_{it}+\beta_{1}\sum_{ij}^{N}w_{ij}EX_{jt}+\alpha_{2}FD_{it}\\ & +\alpha_{3}\ln{\left(ER\right)}_{it}+\alpha_{4}\ln{\left(EC\right)}_{it}\\ & +\alpha_{5}\ln{\left(POP\right)}_{it}+\alpha_{6}ES_{it}+\alpha_{7}UL_{it}+\alpha_{8}UL_{it}{}^{2}+\mu_{i}+\varepsilon_{it} \end{array}$$

As one of the important participatory effects of Chinese economic reform, FD influences FDI and EPEE, so we continue to add the interactive term of FDI and FD for further discussion, as shown in Equations (6)–(8).

(6) $$\begin{array}{cc} EPEE_{it}= & \rho \sum_{ij}^{N}w_{ij}EPEE_{jt}+\alpha_{1}FI_{it}+\beta_{1}\sum_{ij}^{N}w_{ij}FI_{jt}+\alpha_{2}FD_{it}+\alpha_{9}FI_{it}\times FD_{it}+\alpha_{3}\ln{\left(ER\right)}_{it}\\ & +\alpha_{4}\ln{\left(EC\right)}_{it}+\alpha_{5}\ln{\left(POP\right)}_{it}+\alpha_{6}ES_{it}+\alpha_{7}UL_{it}+\alpha_{8}UL_{it}{}^{2}+\mu_{i}+\varepsilon_{it}, \end{array}$$

(7) $$\begin{array}{cc} EPEE= & \rho \sum_{ij}^{N}w_{ij}EPEE_{jt}+\alpha_{1}\ln{\left(SC\right)}_{it}+\beta_{1}\sum_{ij}^{N}w_{ij}\ln{\left(SC\right)}_{jt}+\alpha_{2}FD_{it}\\ & +\alpha_{9}\ln{\left(SC\right)}_{it}\times FD_{it}+\alpha_{3}\ln{\left(ER\right)}_{it}+\alpha_{4}\ln{\left(EC\right)}_{it}\\ & +\alpha_{5}\ln{\left(POP\right)}_{it}+\alpha_{6}ES_{it}+\alpha_{7}UL_{it}+\alpha_{8}UL_{it}{}^{2}+\mu_{i}+\varepsilon_{it}, \end{array}$$

(8) $$\begin{array}{cc} EPEE_{it}= & \rho \sum_{ij}^{N}w_{ij}EPEE_{jt}+\alpha_{1}EX_{it}+\beta_{1}\sum_{ij}^{N}w_{ij}EX_{jt}+\alpha_{2}FD_{it}+\alpha_{9}EX_{it}\times FD_{it}\\ & +\alpha_{3}\ln{\left(ER\right)}_{it}+\alpha_{4}\ln{\left(EC\right)}_{it}+\alpha_{5}\ln{\left(POP\right)}_{it}+\alpha_{6}ES_{it}+\alpha_{7}UL_{it}\\ & +\alpha_{8}UL_{it}{}^{2}+\mu_{i}+\varepsilon_{it} \end{array}$$

In these equations, *i* and *t* stand for the province and year, respectively, *j* represents nearby provinces (*i* ≠ *j*) and $w_{ij}$ is the basic elements of the spatial weight matrix *W*. The other variables are defined as before.

Before estimating the model parameters, the spatial weight matrix *W* needs to be set. Some of the sample provinces are apt to be affected not only by neighboring, but also by non-bordering regions. Compared with the binary contiguity spatial weight matrix, the distance spatial weight matrix is suitable to calculate the spatial correlation. Therefore, this paper uses the reciprocal of geographical distance between different provincial capitals as the spatial weight matrix *W*. The form is as follows:

(9) wij={1dij, (i≠j,i=1,⋯,N;j=1,⋯,N)Wijd=0, (i=j,i=1,⋯,N;j=1,⋯,N).

The Spatial Durbin model captures the effect of spatial lag for the explained variable and explanatory variables jointly. The maximum likelihood method can be applied to solve the endogenous problem effectively and thus, provide the theoretical framework for analyzing the direct and indirect effects of spatial lag values [71]. The direct and indirect effects are calculated as:

(10) $$\frac{\partial_{y_{i}}}{\partial_{x_{ir}}}=\frac{\left(I\beta_{r}+\left({\left(w\right)}_{ii}\theta_{r}\right)\right)}{\left(I-\rho W\right)},$$

(11) $$\frac{\partial_{y_{i}}}{\partial_{x_{jr}}}=\frac{\left(I\beta_{r}+\left({\left(w\right)}_{ij}\theta_{r}\right)\right)}{\left(I-\rho W\right)},\;i\neq j$$

where Equation (10) denotes the direct effect and Equation (11) refers to the indirect effect. $\beta_{r}$ and $\theta_{r}$ denote the coefficient of the *r*th explanatory variable and the spatial lag of the *r*th explanatory variable, respectively.

## 4. Sample Selection and Variable Settings

The sample includes panel data on 30 provincial-level administrative regions in China, which excludes the Tibet Autonomous Region, Macau, Hong Kong and Taiwan Provinces from 2007 to 2016. All data is collected from China Statistical Yearbook, China Environmental Statistics Yearbook, China Environmental Yearbook, China Science and Technology Statistical Yearbook, China Energy Statistics Yearbook, China’s Province Statistical Yearbook and the National Data Network.

### 4.1. Explained Variable

The explained variable is EPEE, which was calculated using the model described above. EPEE is the ratio of regional pollution removal output to government expenditure on environmental protection. The financial input and output data of environmental protection were collected based on the research samples using the aforementioned data sources. Following previous studies [19,20], seven kinds of variables were used. Specifically, the ratio of governmental spending on environmental protection to regional GDP was the input, while the rate of industrial wastewater treatment, industrial sulfur dioxide removed, industrial nitrogen oxide removed, industrial smoke and dust removed, industrial solid waste comprehensive utilization and domestic garbage harmless treatment were the desirable outputs. The outputs in 2016 were industrial solid waste comprehensive utilization and domestic garbage harmless treatment because data of waste gas and wastewater was incomplete in 2016 in the China Environmental Statistics Yearbook and the China Environmental Yearbook. The definitions and descriptive statistics of input–output factors are shown in Table 1.

### 4.2. Explanatory Variables

The explanatory variables included FDI, FD, environmental regulation, the level of economic development, total population, energy consumption structure and urbanization level. FDI was the core explanatory variable, whereby both the quantity of FDI and the quality of foreign investment, in terms of the average scale of FDI and export pull of foreign capital, were considered. FD was a regulating variable and other variables were control variables. All variables were defined and described in Table 2 below.

#### 4.2.1. Core Explanatory Variables

This paper used the quantity and quality of FDI to represent FDI and the quality of FDI was determined by the average scale of FDI and the export pull of foreign capital. The quantity of FDI (which is henceforth referred to as FI), as a percentage of GDP, reflects the openness of each province in the field of international investment.

The quality of FDI was measured as a complex system composed of multiple indicators reflecting different sources, scale and mode of entry [34,73]. Regarding the average scale of FDI (SC), the variable was defined as the result of the expansion of foreign capital, which is conducive to technological innovation and gathering capital and talents. SC also reflects the stronger willingness to engage in FDI. When the scale of FDI increases, there are more opportunities to exchange and learn about management and technologies between domestic enterprises and foreign-funded enterprises. Therefore, SC is one of the indicators to measure the quality of FDI. This paper used the actual amount of FDI divided by the number of foreign-funded enterprises to represent the average scale of FDI and used the logarithmic form in the model. 

In terms of the export pull of foreign capital (EX), FDI is generally conducted by multinational corporations and the export pull of foreign capital is conducive to expanding the export field of multinational companies (i.e., those providing the financing). On the other hand, when multinational corporations train and transfer knowledge to foreign subsidiaries, they will facilitate the diffusion of some proprietary and confidential technology to other companies. This improves the level of technologies of the host country’s enterprises and the ability of their exports to participate in world competition. All in all, the export pull of foreign capital is also an important indicator. This paper uses the ratio of the export value of foreign-funded enterprises on the total export volume of each region to express the export pull of foreign capital.

#### 4.2.2. The Regulating Variable

FD is an important indicator reflecting the extent of fiscal autonomy in each province. FD affects EPEE through local government behavior. The local government’s behavioral activities are mainly reflected in the income and expenditure budget. Therefore, FD can be expressed from the perspective of expenditure, and this paper used the ratio of the per capita financial expenditure in each province’s budget on the per capita financial expenditure in the national budget to express FD. The per capita value applied to eliminate the impact of the size of the population. When the degree of FD is higher, the fiscal autonomy of local governments is greater.

#### 4.2.3. Control Variables

In this paper, five control variables, which affect EPEE, namely, environmental regulation, the level of economic development, total population, energy consumption structure and urbanization level, were included in the model. Environmental regulation (ER) is indicated by the industrial pollution abatement investments of each province. Total population (POP) represents the sum of the population of a province. GDP per capita (EC) represents usually the economic development level of a province. The data could be obtained directly from China’s statistical yearbook. The energy consumption structure (ES) was calculated based on the proportion of coal consumption to the total energy consumption and used the proportion of the urban population to the total population to calculate urbanization level (UL).

## 5. Estimation Results

### 5.1. Status Quo of EPEE

**Figure 2:**
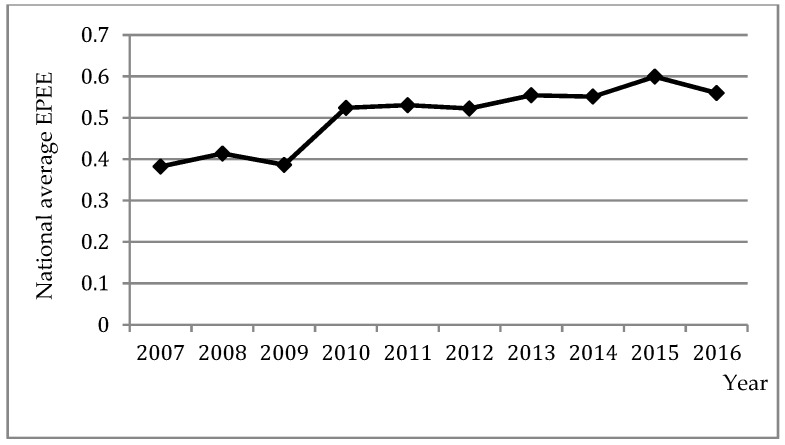
The trend of the national average EPEE from 2007 to 2016.

The trend of the national average EPEE from 2007 to 2016 is shown in Figure 2. The national average EPEE increased by 46.48% in 2016 and 56.78% in 2015 compared with 2007. From 2007 to 2016, EPEE showed an upward trend generally that was in line with the requirements of the government to promote the development of ecological civilization. EPEE in 2009 was the lowest because EPEE of some provinces, such as Jiangsu and Hainan, was much lower than in other years. EPEE in 2016 also fell because the outputs in 2016 were less than in other years.

### 5.2. Test Results

#### 5.2.1. Spatial Autocorrelation Test

To examine whether spatial agglomeration and spatial heterogeneity of variables exist, Moran’s I index was used. Moran’s I of EPEE is shown in Table 3 for every year from 2007 to 2016. Table 3 illustrates that Moran’s I values were positive and significant at the 5% confidence level. This indicates that EPEE presented obvious spatial agglomeration because the constructed statistic follows the standard normal distribution. This was in line with the null hypothesis of spatial independence.

#### 5.2.2. Hausman Test

The result of the Hausman test about the choice between fixed effect and random effect had a negative value. The authors of the Stata software agree that it is a usual outcome for the Hausman test to generate a negative result [74], and mostly the negative result is attributed to the relatively small sample [75]. When using Chinese provincial panel data for testing, the conservative approach is to choose a fixed model because the sample is small [76].

### 5.3. Result Analysis

The empirical results applying the year fixed effect model are presented in Table 4. It is apparent that the spatial correlation coefficients were positive and significant at the 1% level in Model 1, which shows that EPEE would cause spatial dependence on neighboring areas in geospatial space. However, the spatial correlation coefficients were not significant in Model 2 and Model 3, which indicate that the spatial spillover effects came from an exogenous interaction.

As shown in Model 1 in Table 4, the coefficient of FI was positive (0.089) and significant at the 1% confidence level (*z* = 4.50), which means a 1% increase in FI would increase EPEE by 0.089%. On the one hand, this result was in line with the ‘pollution halo’ hypothesis that the increase in FI was conducive to the improvement of environmental quality. With the development of the economy, the government attaches more importance to the development of environmental quality and supervision of foreign-funded projects. The government also restricts and prohibits foreign-funded projects with high pollution and high-energy consumption. At the same time, residents gradually improve their requirements for environmental quality. The government gives higher priority to high technology industries and the introduction of green foreign capital. Local enterprises are driven to carry out green production through cleaner technologies and environmental governance experience under competitive incentives, demonstration effects and diffusion effects. On the other hand, the increase in FI had led to an increase in local government revenues, which reduced the necessity for local governments to reduce the expenditure on environmental protection due to competition with each other. Therefore, FI had a positive impact on EPEE. The coefficient $\beta_{1}$ of the FI spatial lag term was positive (0.570) under the distance spatial weight matrix. Local governments compete with each other for FDI to develop their economy so government competition caused spillover effects and affected other provinces. Increasing environmentally friendly FDI in a certain region may lead other regions to increase their demand for FDI and prefer to choose foreign projects with low energy consumption and low pollution characteristics.

As shown in Model 2 in Table 4, SC was positive (0.057) and significant at the 5% confidence level (*z* = 2.27), which means a 1% increase in scale would increase EPEE by 0.057%. If the dependence on FDI was higher, the local government’s ability to absorb FDI was stronger. SC became larger and it was more likely for foreign enterprises to form economies of scale, which was conducive to the transformation of the industrial structure and the reduction of pollution emissions. What is more, foreign enterprises were more inclined to adopt globally uniform environmental standards and pollution treatment technologies. The coefficient $\beta_{1}$ of the SC spatial lag term was positive (0.440) under the distance spatial weight matrix. With expanding the scale of FDI, the scale of foreign companies increased and the number of employees owned by foreign companies also increased. Thus, it became easier to generate green technology spillovers through the flow of human capital. Therefore, the increase in the scale improved EPEE.

As shown in Model 3 in Table 4, EX was positive (0.273) and significant at the 1% confidence level (*z* = 3.99), which means a 1% increase in export pull would increase EPEE by 0.273% and this was closely related to the export-oriented foreign economic model. For a long time, foreign enterprises mainly focused on export-oriented enterprises. The export pull has helped domestic enterprises to expand to overseas markets and provided opportunities for domestic enterprises to learn and exchange foreign environmental technologies. What is more, the proportion of foreign enterprises’ exports increased and squeezed the profit space of local enterprises to a certain extent. In order to meet the strict environmental standards of foreign countries, local enterprises will be forced to transform and upgrade to improve environmental quality. The coefficient $\beta_{1}$ of the EX spatial lag term was positive (2.168) under the distance spatial weight matrix. Multinational corporations cause technology spillover effects because of export pull. Green technology was exchanged and diffused among the provinces frequently, which was conducive to the increase in pollution treatment capacity and improvement of environmental quality.

The regression coefficient of FD was negative, which indicates that a higher degree of FD results in stronger financial autonomy for local governments and under the interest-driven and single incentive system, there is an incentive for local governments to enter a ‘race to the bottom’ to develop the economy, ignoring the deterioration of environmental quality. When the degree of FD was low, local financial autonomy was weak and local fiscal expenditure would be restricted by central financial subsidies. Local governments are more inclined to obey the central government’s policy arrangements, contributing to reducing environmental pollution and improving EPEE.

Environmental regulation (ER) was positively related to EPEE, which indicates that the increase in investment in industrial pollution treatment was conducive to environmental protection. The regression coefficient of the level of economic development (EC) was positive, indicating that the awareness of ecological protection increased between 2007 and 2016 in China. With the improvement of economic development, local governments spend more on environmental treatment, promote innovation in environmental protection technology and improve the environmental quality. The regression coefficient of population (POP) was negative, which indicates more people lead to greater pressure on environmental protection and lower EPEE. The regression coefficient of the energy consumption structure (ES) was negative, which indicates that a high proportion of coal consumption leads to more serious environmental pollution and lower EPEE. The primary coefficient of urbanization level (UL) was negative and the quadratic coefficient of urbanization level was positive, which indicates that there is a U-shaped relationship between urbanization level and EPEE. Before the turning point, local governments spend more on economic construction and reduce environmental expenditure but after the turning point, local governments pay more attention to environmental problems because of residents’ increasing environmental requirements and use of green technology.

Table 5 presents the detailed results of direct, indirect and total effects based on Model 1–Model 3, which were similar to the corresponding regression results in Table 4. What is more, it confirmed that it was rational to use the spatial model to explore the spatial spillover of FI, SC and EX. To be specific, a 1% growth in FI would directly increase EPEE by 0.103% and indirectly increase EPEE by 0.893% respectively, among the provinces. In terms of the quality of FDI, a 1% increase in SC and EX would directly increase EPEE by 0.058% and 0.273%, respectively, and indirectly increase EPEE by 0.480% and 2.215% among the provinces. This indicates that higher FDI was conducive to beneficial competitive incentives, demonstration effects and diffusion effects in local pollution treatment capacity and EPEE, while lower FDI would induce negative effects.

The regression coefficient and spatial correlation coefficients of EX were more significantly positive than the coefficients of FI and SC. The main reason for this was that EX caused the frequent exchange of green technology and knowledge among the provinces and the spillover effects of the export pull were more obvious than the other two. The regression coefficient and spatial correlation coefficients of SC were the lowest, which indicates that the scale had a weaker impact on the efficiency of government spending on environmental protection because the scale led to slow, long-term effects.

### 5.4. Further discussion of FDI, FD and EPEE

As shown in Table 6, the interactive term of FDI and FD was significant at the 5% level, FI was significant at the 5% level (*z* = −2.47), SC was significant at the 1% level (*z* = −4.08) and EX was significant at the 10% level (*z* = −1.89). This indicates that FD could influence the effect of FDI. The coefficients of FI, SC and EX were positive (0.200, 0.209 and 0.709) and significant at the 5% confidence level (*z* = 4.45, 4.45 and 3.31, respectively). Compared with Table 4, the coefficients of FI, SC and EX increased by 0.111%, 0.152% and 0.436%, respectively, in Table 6. This indicates that FD had a positive impact on the effect of FDI, although FD itself affected EPEE negatively. FD gives local governments financial autonomy and government reduces the expenditure on the environmental protection to cope with economic performance appraisal. However, FD stimulates FDI to play its role when local government has more autonomy and supports the introduction of foreign capital. Under financial centralization, the negative effects of FD became smaller. This suggests that China should adhere to the principle of combining fiscal centralization and decentralization, allocate more expenditure to environmental protection and develop foreign-funded projects to improve EPEE.

### 5.5. Robustness Test

The lag term of FI, SC and EX are regarded as instrumental variables and instrumental variables are conducive to solving the endogeneity problem and the robustness problem [77]. The result of the Hausman test on instrumental variables had a positive value but *p* > 0.1, which means that the result of the instrumental variable regression was not significantly different from the ordinary regression [74]. The spatial generalized moment estimation (SPGMM) was adopted to further solve the endogeneity problem caused by the explanatory variables and error terms to ensure the robustness of the results (Table 7). The coefficient and the trend of each variable and comparison result were similar to the original result.

## 6. Conclusions and Policy Recommendations

This paper constructed a theoretical framework to further explore the relationship and impact mechanisms of FDI, fiscal decentralization and the efficiency of China’s governmental expenditure on environmental protection (EPEE). Based on provincial panel data from 2007 to 2016 in China, the output-oriented DEA scale return model was used to measure the EPEE. Empirical analysis was also conducted based on spatial econometric models. We asked whether FDI promotes or inhibits EPEE. We also asked whether fiscal decentralization plays a moderating role in the relationship between FDI and EPEE. Our study drew the following conclusions: Firstly, EPEE, overall, increased for the 10 years of study, though a decline was observed in 2009 and 2016. Secondly, both the quantity and quality of FDI were positively related to EPEE. The quantity of FDI affected EPEE positively. The effect of increasing the scale of FDI was positive but the effects were weaker than that of the export pull. Overall, the effect of the quality of FDI was stronger than the effect of the quantity of FDI. Thirdly, in terms of spatial effects, EPEE was defined by spatial dependence on neighboring areas in geospatial space. The spatial coefficients for the quantity of FDI, the average scale of FDI and the effects of export pull of foreign capital were positive and significant. This means that an increase in foreign investment will help to enhance EPEE in other cities. There was a significant, negative relationship between FD and EPEE but FD could improve the effect of FDI.

Based on the main conclusions in this paper, some relevant policy suggestions are as follows:

Both the quality and the quantity of FDI should be enhanced. Local governments should actively attract FDI to ensure the availability of governmental funds, which is the basis for all government spending on environmental protection. While expanding the absorption of FDI, local governments should strengthen the supervision of foreign investment projects and constantly update the negative environmental protection list of foreign investment projects. To effectively generate the ‘pollution halo’ effect of foreign investment effectively, local governments should improve foreign investment access standards and guide high-quality and high-efficiency FDI into advanced and high-tech green industries. Local governments should try to reduce high-polluting foreign-funded projects, increase taxation on existing environmentally unfriendly foreign-funded projects and avoid increasing investment in subsequent periods. The investment and utilization value of government environmental protection financial funds will be improved as a result.

As for the spillover effects of FDI, the scale of foreign-funded enterprises is to enhance by absorbing higher levels of FDI, which is conductive to encouraging interaction and cooperation between foreign-funded enterprises and local enterprises. Some enterprises successfully learn in their own way. Local governments can encourage local enterprises to learn advanced environmental technologies and green production experience. Local enterprises can also imitate the establishment of strict and accurate emission standards from more advanced enterprises that are nearby. This will be conducive to positive spatial spillover effects. Then, pollutive emissions are reduced and financial funds from government subsidies for environmental protection are used effectively. The overall promotion of green and healthy sustainable development in the region will increase the utilization rate of government spending on environmental protection.

The central government should improve the supervision system for local governments by, reasonably restraining the power of local governments, encouraging regard for environmental protection as an important assessment standard, strictly enforcing the rules on sewage charges, continuing to carry out environmental protection education, and strengthening scientific and technological research on environmental protection. The central government should vigorously promote clean energy, combined with foreign investment to maximize the effectiveness of local government environmental expenditures.

## Figures and Tables

**Table 1:** Input–output factor definitions and descriptive statistics.

	Definition	Mean	Standard Deviation	Minimum	Maximum	Unit
**Outputs**	The rate of industrial wastewater treatment	0.673	0.104	0.448	0.892	%
	The rate of industrial sulfur dioxide removed	0.600	0.175	0.050	0.874	%
	The rate of industrial nitrogen oxide removed	0.132	0.139	0	0.919	%
	The rate of industrial smoke and dust removed	0.976	0.019	0.894	0.995	%
	The rate of industrial solid waste comprehensive utilization	0.684	0.186	0.299	0.998	%
	The rate of domestic garbage harmless treatment	0.814	0.186	0.230	1	%
**Input**	The ratio of governmental spending on environmental protection to regional GDP	0.007	0.005	0.001	0.036	%

**Table 2:** Definitions and descriptive statistics of all variables in econometric model.

Definition	Variable	Observation	Mean	Standard Deviation	Minimum	Maximum	Unit
Efficiency of governmental spending on environmental protection	EPEE	300	0.50228	0.275341	0.048	1	%
The quantity of FDI	FI	300	0.3710924	0.5376567	0.0473056	5.702378	%
Average scale of FDI	SC	300	42535.54	52554.97	4450.103	403425.6	Ten thousand RMB
Export pull of foreign capital	EX	300	0.3129154	0.2117223	0.0006384	0.7639295	%
Fiscal decentralization	FD	300	5.989259	2.856394	2.307817	14.87644	%
Environmental regulation	ER	300	211155.3	195868.8	3563	1416464	Ten thousand RMB
The level of economic development	EC	300	26412.96	12787.03	7878	62041	Ten thousand RMB
Total population	POP	300	4467.49	2677.044	552	10999	Ten thousand
Energy consumption structure	ES	300	0.9562983	0.3843078	0.1217547	1.991696	%
Urbanization level	UL	300	0.5241235	0.1233595	0.282489	0.8960662	%

**Table 3:** Moran’s I index of environmental protection expenditure efficiency.

Year	Moran’s I	SD (I)	*Z*	*p*
2007	0.400	0.119	3.649	0.000
2008	0.528	0.119	4.714	0.000
2009	0.495	0.119	4.455	0.000
2010	0.665	0.120	5.835	0.000
2011	0.547	0.120	4.840	0.000
2012	0.573	0.121	5.039	0.000
2013	0.457	0.120	4.112	0.000
2014	0.485	0.121	4.308	0.000
2015	0.459	0.120	4.099	0.000
2016	0.209	0.120	2.024	0.043

**Table 4:** The results of the spatial model at the provincial level.

Variable	Model 1	Model 2	Model 3
FI	0.089 *** (4.50)		
Ln(SC)		0.057 ** (2.27)	
EX			0.273 *** (3.99)
FD	−0.078 *** (−4.64)	−0.071 ** (−3.94)	−0.072 *** (−4.27)
Ln(ER)	0.062 ** (2.34)	0.066 ** (2.48)	0.070 ** (2.53)
Ln(EC)	0.469 *** (4.67)	0.392 *** (3.75)	0.299 *** (2.98)
Ln(POP)	−0.085 *** (−2.60)	−0.093 *** (−3.01)	−0.116 *** (−3.72)
ES	−0.176 *** (−4.30)	−0.175 *** (−4.62)	−0.233 *** (−6.07)
UL	−3.263 *** (−3.00)	−2.960 ** (−2.55)	−2.967 *** (−2.66)
UL^2^	2.810 *** (3.39)	2.519 *** (2.85)	2.732 *** (3.13)
ρ	0.321 ** (2.48)	0.069 (0.59)	0.009 (0.06)
W×FI	0.570 ** (2.09)		
W×Ln(SC)		0.440 *** (3.77)	
W×EX			2.168 *** (4.38)
Adj.R^2^	0.9112	0.9181	0.9269
Log like	194.7237	194.0993	201.0897

Note: ***, ** and * represent significance levels of 1%, 5% and 10% respectively; *z* values are shown in parentheses.

**Table 5:** The direct and indirect effects of the spatial Durbin model at the provincial level.

	Model 1	Model 2	Model 3
Variable	Total Effect	Direct Effect	Indirect Effect
FI	0.996 ** (2.21)	0.103 *** (4.35)	0.893 ** (2.05)
Ln(SC)	0.539 *** (4.55)	0.058 ** (2.43)	0.480 *** (4.06)
EX	2.488 *** (5.21)	0.273 *** (3.90)	2.215 *** (4.86)

Note: ***, ** and * represent significance levels of 1%, 5% and 10% respectively; *z* values are shown in parentheses.

**Table 6:** The results of the spatial model including the interaction item at the provincial level.

Variable	Model 4	Model 5	Model 6
FI	0.200 *** (4.45)		
Ln(SC)		0.209 *** (4.45)	
EX			0.709 ** (3.31)
FD	−0.065 *** (−4.39)	0.190 *** (2.99)	−0.063 *** (−4.27)
Ln(ER)	0.058 ** (2.37)	0.053 ** (2.33)	0.051 ** (2.17)
Ln(EC)	0.480 *** (5.66)	0.473 *** (5.32)	0.380 *** (5.18)
Ln(POP)	−0.071 ** (−2.32)	−0.076 *** (−3.19)	−0.125 *** (−4.94)
ES	−0.182 *** (−4.51)	−0.170 *** (−4.62)	−0.214 *** (−8.52)
UL	−4.561 *** (−5.67)	−6.168 *** (−6.17)	−5.309 *** (−3.61)
UL^2^	4.040 *** (6.70)	5.225 *** (6.89)	4.848 *** (3.68)
FI×FD	−0.025 ** (−2.47)		
Ln(SC)×FD		−0.025 *** (−4.08)	
EX×FD			−0.078 * (-1.89)
ρ	0.443 *** (3.75)	0.243 ** (2.01)	0.034 (0.21)
W×FI	0.520 * (1.93)		
W×Ln(SC)		0.430 *** (3.66)	
W×EX			2.256 *** (4.85)
Adj.R^2^	0.9162	0.9290	0.9471
Log like	200.3963	214.9849	211.5132

Note: ***, ** and * represent significance levels of 1%, 5% and 10% respectively; *z* values are shown in parentheses.

**Table 7:** The results of spatial generalized moment estimation (SPGMM).

Variable	Model 1	Model 2	Model 3	Model 4	Model 5	Model 6
FI	0.081 *** (4.51)			0.230 *** (5.81)		
Ln(SC)		0.035 ** (1.97)			0.204 *** (6.77)	
EX			0.208 *** (3.73)			0.631 *** (5.93)
FD	−0.052 *** (−6.66)	−0.056 *** (−6.92)	−0.052 *** (−6.31)	−0.041 *** (−4.92)	0.220 *** (5.25)	−0.042 *** (−5.06)
Control variables	Yes	Yes	Yes	Yes	Yes	Yes
ρ	0.364 *** (2.73)	0.356 *** (2.61)	0.282 ** (1.99)	0.407 *** (3.17)	0.405 *** (3.18)	0.351 *** (2.60)
W×FI	0.390 *** (3.38)			0.395 * (1.94)		
W×Ln(SC)		0.211 ** (2.24)			0.030 (0.23)	
W×EX			1.324 *** (3.45)			2.221 *** (3.66)
FI×FD				−0.035 *** (−4.14)		
Ln(SC)×FD					−0.027 *** (−6.69)	
EX×FD						−0.080 *** (−4.77)
F- Test	70.7050	64.2622	69.3353	67.4151	67.5525	66.2215
Log-L	205.6023	196.1053	202.6035	214.4127	217.1004	214.1614
R^2^	0.9514	0.9476	0.9507	0.9541	0.9541	0.9534
Obs	300	300	300	300	300	300

Note: ***, ** and * represent significance levels of 1%, 5% and 10% respectively; *z* values are shown in parentheses.
